# Development and evaluation of a triplex real-time PCR assay for enhanced plague diagnostics in Madagascar

**DOI:** 10.1371/journal.pntd.0013278

**Published:** 2025-07-24

**Authors:** Beza Ramasindrazana, Zoé A. Rahalison, Philippe Gauthier, Guillain Mikaty, Zaina I. Bodoarison, Lanto A. Maminirina, Soloandry Rahajandraibe, Mamy G. Randriamanantsoa, Gilbert Kayoko, Jean-Claude Manuguerra, Anne-Sophie Le Guern, Andriamiliharison J. Rasamindrakotroka, Minoarisoa Rajerison

**Affiliations:** 1 Plague Unit, Institut Pasteur de Madagascar, Antananarivo, Madagascar; 2 Faculté des Sciences, Université d’Antananarivo, Antananarivo, Madagascar; 3 Faculté de Médecine Humaine, Université d’Antananarivo, Antananarivo, Madagascar; 4 CBGP, IRD, INRAE, CIRAD, Institut Agro, Université de Montpellier, Montpellier, France; 5 Institut Pasteur, Université Paris Cité, Unité Environnement et Risques infectieux, Cellule d’Intervention Biologique d’Urgence, Paris, France; 6 Ministry of Public Health, National Plague Control Program, Antananarivo, Madagascar; 7 World Health Organization, Emergency Preparedness and response, Antananarivo, Madagascar; 8 Institut Pasteur, Université Paris Cité, Yersinia Research Unit, National Reference Centre for Plague and other yersiniosis, Paris, France; Yale University School of Medicine, UNITED STATES OF AMERICA

## Abstract

**Background:**

The plague, caused by *Yersinia pestis*, remains a critical public health issue, particularly in endemic regions like Madagascar. Rapid and accurate detection of this pathogen is essential for effective outbreak management and timely intervention. Following the urban plague outbreak of 2017, a new molecular diagnostic algorithm was developed and introduced into routine use. However, certain cases required combining real-time and conventional polymerase chain reaction (PCR) methods. While effective, this approach often delayed obtaining conclusive results, an issue that can hinder swift outbreak responses. The aim of this study is to design and optimize a three-target real-time PCR assay (qPCR) for the detection of *Y. pestis* in clinical samples.

**Methods:**

The assay targeted three genes: *caf1*, *pla*, and *yopM*, located on the plasmids pMT1, pPCP1, and pCD1, respectively. Conducted at the Institut Pasteur de Madagascar (IPM), the study evaluated the assay using both pure bacterial cultures and clinical samples, including 50 bubonic aspirates and 50 respiratory specimens.

**Results:**

Using bacteriology technique as the reference standard, the triplex qPCR demonstrated a sensitivity of 100% (89–100%) and a specificity of 82%. The positive predictive value (PPV) was 73% and the negative predictive value (NPV) was 100% (91–100%). The coefficient of agreement kappa was 0.74, with a p-value of <0.0001.

Notably, the new assay resolved 100% of previously inconclusive cases from the duplex qPCR test targeting only *pla* and *caf1*.

**Discussion:**

While a new plague diagnostic algorithm has been set up after the outbreak in 2017, the present study suggests a real-time PCR assay based on three genes to improve the speed and accuracy of plague diagnostic. Furthermore, this new technique is a valuable tool for managing plague outbreaks and supporting field diagnostics not only in Madagascar but also in countries with plague.

**Conclusions:**

The developed triplex assay to molecularly diagnose *Y. pestis* in human samples improves the standard already in place and allows to resolve ambiguities previously associated with inconclusive results from duplex qPCR tests, thereby reinforcing the reliability and accuracy of this new technique. Implementing this new method into routine will enable a faster, more effective response to plague outbreaks by reducing the time needed to confirm plague cases and limiting the spread of the diseases. This new technique is also flexible and can be undertaken close to human cases with adequate biosecurity and biosafety measures.

## Author’s summary

Plague, a severe infectious disease caused by the bacterium *Yersinia pestis*, remains a significant public health concern in Madagascar, where seasonal outbreaks continue to occur. Rapid and accurate diagnosis is critical for timely treatment and outbreak control. Current diagnostic methods, including bacteriology and rapid diagnostic tests, often face limitations in speed, sensitivity and/ or specificity especially in remote settings. In this study, we developed and evaluated a new molecular diagnostic tool: a triplex real-time PCR assay that simultaneously detects three genes from *Y. pestis* (*pla*, *caf1*, and *yopM*). This approach enhances diagnostic accuracy while reducing turnaround time. The assay was validated using clinical samples collected in Madagascar and showed high sensitivity (100%) and negative predictive value (100%), outperforming conventional techniques. It is important to notice that this technique resolved previously inconclusive results produced by standard molecular methods. Our findings demonstrate that this triplex qPCR is a robust, rapid, and reliable diagnostic tool that can improve plague detection, especially in outbreak settings. Moreover, its implementation could significantly strengthen public health responses and laboratory capacities in endemic regions like Madagascar.

## Introduction

Plague, an anthropozoonotic bacterial disease caused by *Yersinia pestis*, a Gram-negative bacillus, has been responsible for three major pandemics, including the Black Death in the 14th century, which devastated Europe [1,2]. The bacterium can persist naturally in sylvatic cycles involving small mammals, particularly rodents, and their environment. Sporadic epizootics can lead to widespread infections among small mammals, rodents, and their fleas, facilitating transmission to incidental hosts, including humans. Humans can contract plague through flea bites, direct contact with tissues or fluids from infected animals, or inhalation of infectious droplets [3–6].

In the case of human plague, there are two main clinical forms: bubonic and pneumonic depending on the route of transmission. Bubonic plague is the most common form and is typically acquired through the bite of an infected flea [7]. Pneumonic plague, although less common, occurs through inhalation of infectious aerosols during human-to-human transmission or as a secondary complication of bubonic plague via hematogenous spread to the lungs [8]. Pneumonic plague can spread rapidly in situations involving close contact and interaction between individuals, with case fatality rates reaching nearly 100% if left untreated. However, timely diagnosis and treatment within 24 hours of symptoms onset lead to high recovery rates [8,9].

Since the third pandemic, plague outbreaks have persisted in certain regions around the world, particularly in Africa, Asia, and the Americas. Over 98% of reported cases and deaths have occurred in five countries: the Democratic Republic of the Congo (DRC), Madagascar, Uganda, the United Republic of Tanzania and Peru [10]. In Madagascar, cases of human plague are reported nearly every year during the seasonal outbreak running from September to April. Madagascar has a long story with plague, first introduced in 1898 through trade routes. Since then, outbreaks have been common particularly in endemic foci [11,12]. Two main foci were acknowledged with the central and northern highlands above 800 meters in altitude, this distribution is linked to the presence of vector fleas, which are less abundant and absent below 800 meters [13,14]. Additionally, a third plague focus emerged outside the central and northern highlands, in the sea port city of Mahajanga, where plague first appeared in 1902 [15–18].

In 2017, Madagascar experienced a severe urban plague outbreak, underscoring the critical need for rapid diagnostic tools [19]. This pneumonic plague outbreak affected over 2,400 individuals, highlighting the necessity of improved diagnostic techniques for effective crisis management [20]. Identification of *Y. pestis* by bacteriological means (microscopy, culture) remains the gold standard for plague diagnosis, although these methods are time-consuming and not always available during outbreaks, and present health risk for laboratory staff due to the virulence of the bacteria *Y. pestis*. Since plague primarily occurs in remote rural areas, the need for a rapid diagnostic test (RDT) at primary healthcare facilities was recognized early on as an essential tool. RDT based on the Fraction 1 (F1) antigen (F1RDT) of *Y. pestis* was introduced in the early 2000s to enhance diagnostic capacity in field settings [21]. However, these tests often required confirmatory testing through conventional bacteriological methods especially for pneumonic cases as sputum samples can vary widely in quality [21,22]. At that time the molecular biology techniques were used for research, confirmation of isolated strains, and post-endemic investigations [23]. In 2017, the pneumonic plague outbreak created a pressing need for rapid and specific diagnostic tools, leading to the adoption of PCR-based diagnostics. These methods focused on real-time PCR (qPCR) targeting two *Y. pestis* genes: *pla* and *caf1*. To resolve cases with discordant results - such as single-gene positivity in qPCR - an additional conventional PCR assays targeting three genes: *pla*, *caf1*, and *inv1100* (a gene shared by the three pathogenic *Yersinia* but presenting a specific insertion in *Y. pestis*) were introduced for confirmatory testing [20,22,24,25].

In this context, the introduction of the new PCR algorithm for the molecular diagnosis of plague represented a significant advancement in plague diagnostics, aligning with the WHO guidelines for the detection of *Y. pestis* DNA using species-specific PCR methods. By targeting multiple genes in a single reaction, the proposed approach enhances diagnostic accuracy while minimizing the risk of false positives and negatives. However, for discordant results, the additional confirmatory PCR partially impair this gain. During an outbreak, it is crucial to have the result of the diagnostic as soon as possible for the health care staff to provide adequate treatment and response.

Therefore, we hypothesize that a triplex qPCR assay could compensate for the need of confirmatory PCR and would improve the accuracy, sensitivity, and reliability of plague diagnostics compared to dual-target PCR assays.

In this study, we developed a multiplex real-time PCR assay targeting three genes (*caf1*, *pla*, and *yopM*) to detect *Y. pestis* to enhance diagnostic accuracy and reliability. The *caf1* gene, located on the pMT1 plasmid, encodes the Fraction 1 (F1) capsular protein, a widely used diagnostic marker due to its high expression and role in bacterial virulence, as it is a key component in the immune evasion of *Y. pestis* [26]. The *pla* gene, found on the pPCP1 plasmid, encodes the Pla protease, a fibrinolysin crucial for bacterial dissemination by facilitating systemic infection through the degradation of host fibrin clots, making it a common target in PCR-based detection [26,27]. The *yopM* gene, located on the pCD1 plasmid and shared by the three pathogenic *Yersinia*. *YopM* encodes an effector protein involved in the type III secretion system, a key virulence mechanism that allows *Y. pestis* to evade the host immune response and modulate cellular signalling [28]. The conjugated detection of these three plasmids is highly specific of pathogenic *Y. pestis* and can act as fingerprint for the presence of the plague bacillus.

## Materials and methods

### Study design

The study was conducted at the Plague Unit at the Institut Pasteur de Madagascar (IPM), a national reference laboratory for plague diagnostics and a WHO Collaborating Centre for plague research and control. Materials used included clinical samples randomly selected between 2022 and 2023, pure *Y. pestis* strains as well as various reagents required for DNA extraction and PCR amplification.

### Targeted genes

The triplex qPCR assay was designed to detect three genes located on three different plasmids of *Y. pestis*: *caf1* (pMT1), *pla* (pPCP1), and *yopM* (pCD1). The *caf1* and *pla* genes are well-known markers for *Y. pestis*, while *yopM* was included as an additional marker to enhance diagnostic reliability and based on its role in encoding a protein involved in the pathogenesis of *Yersinia* species. Primers and probes for *caf1* and *pla* genes were already available within the Plague Unit (Central Laboratory for Plague).

### Bacterial strain and clinical samples

*Yersinia pestis* strain 6/69 was selected from the biobank of the Plague Unit at Institut Pasteur de Madagascar. This strain was preserved at -80°C in tryptone casein soya (TCS) medium with glycerol for the optimization.

One hundred clinical samples from suspected plague patients (50 bubo aspirates, 50 sputum samples) received at Institut Pasteur Madagascar were used for the evaluation of the triplex qPCR technique.

Additionally, to ensure specificity, other bacterial strains such as *Mycobacterium tuberculosis, Rickettsia* sp., *Bartonella* sp., *Leptospira* sp*.*, *Clostridium difficile*, together with microbial community DNA (Microbial Community DNA Standard, ZymoBioMICS, Zymo Research, S1 Data) were included to verify that neither false-positive amplification nor amplification of non-target organisms occurred.

### DNA isolation

The bacterial strain of *Y. pestis* was sub-cultured in brain-heart infusion broth at 26˚C for 48h. The culture (1.5 ml) was then subjected to DNA extraction using DNeasy Blood & Tissue Kit (Qiagen, Hilden, Germany) according to the manufacturer’s protocols. DNA extracts from clinical samples were obtained using the Qiagen QIAamp DNA Mini Kit (Qiagen, Hilden, Germany). Finally, the purified DNA was eluted in 50µl of low-salt buffer and stored at -20°C until further use. For the other bacteria used to check specificity, DNA extracts were already available in the Unit.

### Alignment of *yopM* gene sequences

Multiple *yopM* gene sequences *Yersinia pestis* were retrieved from the NCBI nucleotide database (https://www.ncbi.nlm.nih.gov/nucleotide/). The sequences were obtained from different strains of the bacterium. These sequences were aligned using the bioinformatics tool MEGA XI [29], utilizing the ClustalW multiple alignment tool. The objective was to identify conserved regions within the gene from which primers and probes were selected using BioEdit Software. More than 40 sequences isolated from strains collected across Africa, America, Europe, and Asia available on NCBI were selected to optimize the sensitivity, accuracy and specificity of the primers and probes design.

### Primer and probe selection and design

The primers and probes used for the detection of the *pla* and *caf1* genes in this triplex qPCR assay were adapted from previously published studies to ensure optimal sensitivity and specificity. The *pla* gene primers and probe were derived from the works of Loïez *et al*. (2003) and Stewart *et al*. (2008) [27,30]. Similarly, the primers and probe for the *caf1* gene, encoding the Fraction 1 (F1) capsular antigen, were adapted from Stewart *et al*. (2008) and Woron *et al*. (2006) [26,30].

Specific primers and probes targeting the *yopM* gene were designed using the Eurofins Genomics Primer Design Tool and LightCycler Probe Design Software 2.0 (Roche Diagnostics) based on aligned gene sequences, following established criteria for primers and probes used in qPCR. Their specificity was verified using BLAST in the NCBI database to ensure accurate in silico evaluation prior to experimental application.

Seven primer pair combinations and one probe targeting *yopM* were designed and tested in simplex PCR before being implemented in triplex PCR to select the most efficient, specific, and sensitive primer set. This preliminary testing ensured that the chosen primers exhibited optimal performance in terms of amplification efficiency, specificity to the target sequence, and sensitivity. Primers and probes designed for *yopM* gene are listed in Table 1.

**Table 1:** Designed primer and probe combinations for *yopM* gene.

Combination	Primer/probe designation	Nucleotide sequence	Size (pb)	%GC	Tm
C1	yopM-F1	GGAATGGGAACGAAATGCC	19	52.6	60.5
	yopM-R1	AAGAGAATTACATGACGCCAC	21	42.9	61.1
C2	yopM-F1	GGAATGGGAACGAAATGCC	19	52.6	60.5
	yopM-R2	GAATTACATGACGCCACTAAAC	22	40.9	59.8
C3	yopM-F2	TGGTCGGAATGGGAACG	17	58.8	60.5
	yopM-R3	ACTCTCTAAATGCGGAGGTA	20	45	60.2
C4	yopM-F2	TGGTCGGAATGGGAACG	17	58.8	60.5
	yopM-R4	GCCACTAAACTCTCTAAATGCG	22	45.5	60.2
C5	yopM-F2	TGGTCGGAATGGGAACG	17	58.8	60.5
	yopM-R5	TAAACTCTCTAAATGCGGAGGTAA	24	37.5	60.3
C6	yopM_F2	TGGTCGGAATGGGAACG	17	58.8	60.5
	yopM-R7	ATGACGCCACTAAACTCTC	19	47.4	60
C7	yopM-F3	GGAATGGTGAACAGAGGG	18	55.6	59.7
	yopM-R6	GAGAATTACATGACGCCACT	20	45	59.8
yopM probe	yopM-Probe	TTGCCTGGACCGACAAGCC	19	63.2	65.1

Primers and probe were provided in lyophilized form by Eurofins Genomics and reconstituted with Tris-EDTA following the quantity specified by the supplier, then stored at -20°C for preservation. The concentration of the stored primers and probes is 100µM.

Fluorophores were chosen for utilization with the MyGo Pro thermocycler to avoid spectral overlaps in analysis. The dye-target combinations are as follows: 6-carboxyfluorescein (6-FAM) for *caf1*, Cyanine 5 (Cy5) for *yopM*, and LightCycler Red 610-N-hydroxysuccinimide ester LSR (Red610) for *pla*.

### Simplex assay for YopM Primer

The first step in developing the assay involved testing different combinations of primers and probes independently in qPCR to select the best combination for multiplexing with the two other genes (*pla* and *caf1*).

All reactions were carried out using the MyGo Pro thermocycler (IT-IS International Ltd (Novacyt Group)) and the LightCycler480 Probes Master reagent (Roche Diagnostics). Each reaction included 10 µl of LC480 Probes Master, 5 µl of DNA extract (diluted to 1/10^th^), 0.1 µl of *yopM* probe for a final concentration of 0.25 µM, 0.2 µl of primers for a final concentration of 0.5 µM, and water to a final volume of 20 µl, prepared in duplicate. Amplification was programmed using the MyGo Pro PCR Software 3.4, with a profile consisting of pre-incubation at 95°C for 10 minutes, followed by 50 cycles of 95°C denaturation for 20 seconds, 60°C annealing for 1 minute, and cooling at 40°C for 30 seconds, with fluorescence measured in the corresponding fluorophore channel. Sterile distilled water was used as a negative control, and other pathogens were tested to ensure no cross-reaction occurred.

The PCR results were analyzed and controlled through the MyGo Pro PCR Version 3.4 software, with several criteria used to select the best primer pair. A positive result was identified when the amplification curve displayed a typical sigmoidal shape, and negative results were consistent with the profiles of the negative controls. The efficiency of the PCR process, which measures how effectively DNA is amplified, was also evaluated. This involves calculating how the amount of DNA doubles with each cycle, although this perfect doubling rarely happens in practice. The study measured the reaction’s efficiency using a 10-fold DNA dilutions series and plotting the Ct (Cycle threshold) values against DNA concentrations to ensure accurate results. An acceptable efficiency is between 90% and 110%. Moreover, the sensitivity of the assay was evaluated by determining the lowest detectable amount of DNA, using serial dilutions to establish the detection threshold. Finally, the specificity of the primers was confirmed by ensuring they exclusively amplified the target gene without any cross-reactions or nonspecific amplification, as validated with negative controls. Therefore, the primer pair that yielded the best results based on the analyzed criteria was selected for multiplexing with the *caf1* and *pla* genes and subsequently tested on human clinical samples.

### Real-time triplex assay

The reaction mixture for the triplex assay included the components already mentioned for the simplex assay and incorporated the best primer-probe combination for *yopM*, along with *caf1* and *pla*, each labelled with distinct fluorophores (Cy5, 6-FAM, and Red610 respectively), following the same thermal cycling protocol as the simplex assay.

To optimize the reaction, concentrations were adjusted for compatibility in simultaneous amplification, with probe and concentrations tested between 0.125 and 0.5 µM to leverage each fluorophore’s unique properties, ensuring balanced and specific detection, along with high specificity, sensitivity, and reproducibility. To further enhance PCR amplification, the temperature was adjusted to 56°C for better efficiency, and the integration time was extended by two seconds to improve signal detection, particularly for the lower-intensity Cy5 marker compared to the other fluorophores. DNA samples from pure strain of *Y. pestis* were used as positive control and DNase free ddH_2_O was added to the negative control tubes to check any contamination or primer dimer.

### Sensitivity testing and specificity assessment

The analytical sensitivity was assessed using serial dilutions of *Y. pestis* DNA from 10^-1^ to 10^-6^ in duplicate to determine the lowest detection threshold for each primer pair, identifying the smallest dilution at which the PCR still produced a detectable signal and the limit of detection of the multiplex qPCR assay was also established based on CFU/ml from pure *Y. pestis* strains. Specificity was confirmed by ensuring that no cross-reactions occurred between the different target genes or with other bacteria. Analytical specificity ensured that the primers amplified only the target gene without generating non-specific amplification and confirmed that there was no undesirable interaction between probes and primers. This was verified using negative controls and other pathogens listed previously.

### Evaluation of the assay

The triplex qPCR technique was evaluated using a retrospective series of samples from suspected plague patients, including 100 human samples randomly (bubo aspirates and sputum) selected from the database among those with complete results from biological tests commonly employed for plague diagnosis, collected between 2022 and 2023 (S2 Data).

The results of the triplex qPCR were compared to the conventional diagnostic methods used in Madagascar, including bacteriology, F1RDT, and duplex qPCR *pla-caf1*. A result is considered positive when two or three out of the three genes (*pla*, *caf1*, and *yopM*) are detected. A negative result is defined as the absence of signal for all three genes or the detection of only one out of the three genes, which is considered inconclusive and requires further confirmation. The analysis included samples from buboes and sputum, and the data were systematically organized in an Excel table. This table included patient identification, clinical details, sample information, and the results from routine tests and the new diagnostic technique. All statistical analyses were conducted using R software version 4.3.0 [31].

The evaluation focused on comparing the effectiveness of the triplex qPCR with traditional methods. Sensitivity and specificity were measured to determine the test’s ability to correctly identify both positive and negative cases, respectively. Positive predictive value (PPV) and negative predictive value (NPV) were calculated to assess the likelihood of true positive and true negative results. Cohen’s Kappa coefficient was used to measure the concordance between methods.

The triplex qPCR was also compared to F1 strip tests and duplex qPCR *pla-caf1*, examining overall agreement and repeatability. Repeatability was tested by analyzing the same sample three times using the same conditions and the same thermocycler, while reproducibility was assessed under varying conditions, such as different operators and reagent lots, with variability measured using the coefficient of variation.

### Ethical statements

The DNA samples utilized in this study originated from *Y. pestis* cultures or human biological specimens, which were initially collected by the Central Laboratory for Plague, at IPM which is part of the National Plague Control Program. This program requires mandatory reporting of all suspected human plague cases and the corresponding collection of clinical samples. As these samples, including derived cultures or extracted DNA, were obtained under this obligatory reporting system, they were not classified as human subject research. Furthermore, all isolates and biological specimens were anonymized by removing any identifiable patient information prior to analysis. Consequently, ethical approval from the Malagasy Ethical Committee was not required for this study.

## Results

### *YopM* primer design and selection

**Fig 1:**
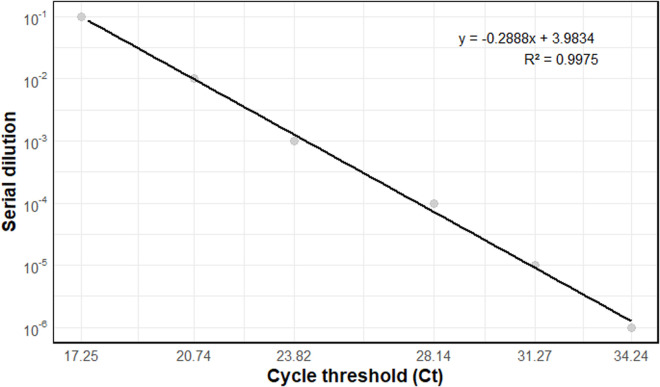
Standard curve for the yopM gene using a 1/10 serial dilution with the combination of primer C7.

Before developing the triplex qPCR, simplex qPCR was conducted for each primer combination targeting the *yopM* gene. Among the seven combinations tested, the combination labelled C7 consistently demonstrated superior performance (Table 2). It achieved a 1.94 (Fig 1) efficiency in the simplex reactions, making it the most reliable option for multiplexing with the *caf1* and *pla* genes. This preliminary testing ensured that the optimal primer set was selected for the subsequent multiplex qPCR assay aimed at detecting *Y. pestis*. Primer and probe sequences for each gene retained for the optimization and evaluation are listed in Table 2.

**Table 2:** Primer and probe sequences for *Y. pestis* qPCR triplex assay.

Primers or probes	Gene (plasmid)	Nucleotide sequence	References
pla F pla R pla Probe	*pla* (pPCP1)	CGAAAGGAGTGCGGGTAATA AATAACGTGAGCCGGATGTC LC610-ATATTGGACTTGCAGGCCAG-BHQ2	[27,30]
caf1 F caf1 R caf1 Probe	*caf1* (pMT1)	ATCGCCATTGCATTATTTGG CCTGTTTTATAGCCGCCAAG 6-FAM-TTAACTGCAAGCACCACTGC-BHQ1	[26,30]
yopM F (C7) yopM R (C7) yopM Probe	*yopM* (CD1)	GGAATGGTGAACAGAGGG GAGAATTACATGACGCCACT Cy5-TTGCCTGGACCGACAAGCC-BHQ3	This study

### Real-time triplex PCR assay

**Fig 2:**
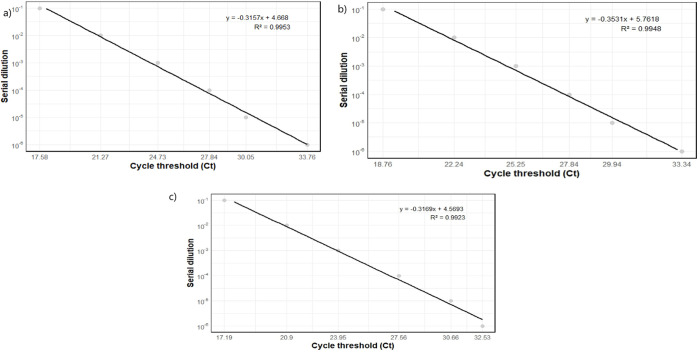
Amplification standard curve of each target with DNA dilutions, Ct values were correlated to the DNA concentration for each target. **a)** *caf1*, **b)** *pla*, **c)** *yopM*.

In the triplex assay using primer set C7 for the gene *yopM*, the Ct values for each gene exhibited a consistent increase with each dilution step as shown in Fig 2. The combination labelled C7 showed consistent performance, with a regular increase in Ct values for the *yopM*, *caf1*, and *pla* genes as the dilution increased as shown in Table 3, indicating good sensitivity of the reaction. The Ct values followed a consistent progression with dilution, and the calculated slopes for the three genes were close to -0.32, suggesting similar reaction efficiency across all targets. The coefficients of determination (R²) were high, confirming the reliability of the reaction. The calculated efficiencies were 2.07 for *yopM* and 2.06 for caf1, while for *pla* it was slightly higher at 2.25.

**Table 3:** Primers and probe final concentrations selected after the optimization (initial concentration was 50 µM).

Primers and probes	Final concentration
yopM F	0.5 µM
yopM R	0.5 µM
caf1 F	0.25 µM
caf1 R	0.25 µM
pla F	0.5 µM
pla R	0.5 µM
yopM Probe	0.25 µM
caf1 Probe	0.125 µM
pla Probe	0.25 µM

### Optimization of primer and probe concentration

Different optimizations have been made regarding the primer and probe concentrations to ensure efficient amplification and detection of the *caf1*, *pla*, and *yopM* genes in the triplex qPCR assay and the final concentrations used are presented below (Table 3).

### Validation of the triplex qPCR assay

The DNA extracts were obtained from a pure strain of *Y. pestis* at different concentrations, ranging from 8.6 x 10^7^ to 1 CFU/ml, and were tested using the optimized triplex qPCR. Amplification was observed even with a preparation containing 1 CFU/ml, thus establishing the detection limit at 1 CFU/ml. Additionally, no cross-reactivity was detected with the negative controls and the other bacteria previously mentioned.

The results of the repeatability test showed an excellent consistency across the three genes. For the *yopM* gene, the coefficients of variation (CV) are very low, ranging from 0.01 to 0.02, even at the lowest DNA concentrations, indicating a highly reliable performance. Similarly, *caf1* also exhibited strong repeatability with consistently low CVs across all concentrations. The *pla* gene showed a slightly higher CV of 0.03 at the highest concentration but remained within an acceptable range for repeatability. In terms of reproducibility, the *yopM* gene had a mean Ct value of 29.356 with a CV of 0.016, demonstrating stability when performed by different operators. The *caf1* and *pla* genes displayed similar reproducibility, with CVs of 0.014 and 0.019, respectively, indicating that the method performed reliably across different settings.

### Performance of the triplex qPCR

We compared the results of the triplex qPCR assay for plague with those obtained from bacteriological culture and the F1RDT, across different clinical forms. In clinically diagnosed bubonic plague cases (n = 50), the triplex qPCR detected 23 positives and 27 negatives. It showed strong concordance with bacteriology, with only one discordant result: the triplex qPCR returned a positive result while culture was negative, suggesting a potential specificity issue. For pneumonic plague cases, the triplex qPCR identified 21 positives and 29 negatives, whereas culture detected only 10 positives. A total of ten discordant results were observed between the two methods. Overall, the concordance rate between triplex qPCR and culture was 89%, with a sensitivity of 100% (95% CI: 89–100%) and a specificity of 82% (95% CI: 71–91%). The positive predictive value (PPV) was 73%, and the negative predictive value (NPV) was 100%. Cohen’s Kappa coefficient was 0.74 (p < 0.0001), indicating substantial agreement.

Compared to the F1RDT, the triplex qPCR assay showed a concordance rate of 97%, with a positive agreement of 96% and a negative agreement of 97%. Cohen’s Kappa was 0.93 (p < 0.0001). Discordant results were found in three cases one bubonic and two pneumonic plague cases.

Although the triplex qPCR assay was not initially designed for a direct comparison with the duplex assay, it helped resolve two previously inconclusive cases, reclassifying one as positive and the other as negative. However, four samples (IDs 8, 30, 75, and 76) that tested positive with the duplex assay yielded negative results with the triplex qPCR in the first experiment. To further investigate these discrepancies, we re-extracted DNA from these samples and retested them using the triplex qPCR after a 1:10 dilution, in accordance with our internal protocol. Three (IDs 8, 30, and 76) out of the four samples then tested positive, while sample ID 75 remained negative.

## Discussion

In an effort to improve the diagnostic tools for plague, our study focused on developing and validating a triplex qPCR assay for the detection of *Y. pestis* in clinical samples. This work aimed to optimize existing molecular methods by incorporating the *yopM* gene into the multiplex assay, along with the established *caf1* and *pla* genes, both of which are well-recognized for their roles in the pathogenicity of *Y. pestis* [28,32,33]. While the *pla* gene is often targeted due to its high sensitivity [34], it poses a risk of false positives due to homologs present in other bacteria [35]. To address this, we introduced the *yopM* gene, previously utilized by Tsukano *et al.* for *Y. pestis* detection [28]. However, adapting *yopM* primers for real time PCR required modifications, as the original primers were designed for conventional PCR.

Our results demonstrated high sensitivity, detecting bacterial concentrations as low as 1 CFU/ml. In comparison to bacteriology, our method achieved a sensitivity of 100% and a specificity of 82%, slightly lower than the 100% specificity reported by Bai *et al*. in pure cultures [32]. The lower specificity in our study may be attributed to the use of clinical samples collected in field conditions, where variables such as sample transport, contamination, or prior antibiotic treatment can affect *Y. pestis* culture outcomes [36]. Notably, several samples deemed negative by bacteriology tested positive using the immunochromatographic F1 antigen test, further emphasizing the robustness of molecular techniques that remain unaffected by such external variables. Statistical analysis showed an overall concordance of 94% between the F1 test and our multiplex PCR, with a kappa value of 0.76 when compared to bacteriology, indicating the reliability of our assay.

Although our study successfully addressed uncertain cases detected by the duplex qPCR *pla-caf1*, certain limitations persist. Our method relies exclusively on plasmid-encoded genes, which prevents the detection of *Y. pestis* strains that may have lost these plasmids, as previously noted by Chanteau *et al*. [21] and Eppinger *et al*. [37]. Additionally, the absence of an internal control in the amplification process represents a significant weakness, as it leaves the assay vulnerable to inhibitors in complex clinical samples, potentially compromising the detection of target genes [24]. The inclusion of an internal control or an additional target, such as the 16S ribosomal RNA gene, could help mitigate this issue. However, doing so would increase costs and heighten the risk of cross-contamination.

Another limitation identified in this study was the discrepancy observed in four clinical samples that tested positive by duplex qPCR but negative with the triplex multiplex assay. While some of these samples were highly positive by F1RDT (highly positive), suggesting a very high bacterial load, others showed lower and lead to inconclusive results. According to our internal protocol, samples suspected of having high DNA concentrations are routinely diluted 1:10 or 1:100 prior to duplex qPCR testing; however, they were tested undiluted with the triplex multiplex assay. Thus, after a new DNA extraction and in accordance with our internal protocol, three out of the four samples were positive, the fourth samples remained, likely due to the number of extractions it had undergone as part of various research projects which may have led to DNA degradation or depletion.

Despite these challenges, the triplex qPCR assay offers a significant advancement in the rapid and accurate detection of *Y. pestis,* making it a valuable tool for improving plague outbreak management, especially in public health emergencies where timely diagnosis and intervention are crucial.

The triplex qPCR significantly reduced diagnostic time compared to traditional bacterial culture methods. While culture typically requires 48–72 hours to yield results, the qPCR assay provided definitive results in just 3–4 hours, including time for sample preparation, DNA extraction, and amplification. The triplex qPCR also proved more resolutive compared to the duplex qPCR previously used by solving right away the discordant results without the need of additional time-consuming conventional PCR. This rapid turnaround is particularly important in outbreak situations, where quick decisions are needed to prevent further spread of the plague. Additionally, direct comparisons between qPCR and culture showed that culture struggled to detect early-stage or low-load infections due to insufficient bacterial growth during the 48–72 hours incubation period. In these cases, the triplex qPCR successfully identified *Y. pestis*, highlighting its superior sensitivity and ability to detect the pathogen even at lower concentrations. Furthermore, the technique is easy to implement in the field, offering faster results at a lower cost, making it an efficient tool for plague surveillance and outbreak response.

As a WHO Collaborating Centre for Plague, this kind of improvement not only provides better diagnostic tools but also helps to detect plague cases early and manage them more effectively. It allows for quick action during outbreaks, reducing the risk of the disease spreading. This improvement supports public health teams in tracking and controlling the disease, making it easier to monitor trends in plague-affected areas and improving preparedness for future outbreaks. Overall, it plays an important role in global efforts to prevent and control plague and in line with the WHO proposition for confirmation test improvement.

The triplex qPCR developed in this study is characterized by its ability to target multiple genetic markers *caf1*, *pla*, and *yopM* in a single assay, combining high sensitivity, specificity, and rapid diagnostic capability. These attributes make it a robust tool for advancing the molecular diagnosis of plague. By demonstrating the effectiveness of multiplexing in detecting *Y. pestis* in human samples, this study addresses critical gaps in existing diagnostic methods. Further, this new technique can be used for plague diagnostics by the different countries where the disease remains endemic.

## Conclusion

This new triplex qPCR assay resolves ambiguities previously associated with inconclusive results from duplex qPCR tests, thereby reinforcing the reliability and accuracy of this diagnostic approach. Consequently, integrating these improved techniques into routine diagnostic practices holds great promise for transforming surveillance systems and enabling a faster, more effective response to plague outbreaks. By reducing the time needed to confirm plague cases, these technological advancements enable quicker mobilization of public health resources, which is crucial for controlling and limiting the spread of this potentially deadly disease. Future development of this methodology will include further specificity testing for the primers. The introduction of new genetic targets, including chromosomal and plasmid genes specific to *Y. pestis* as well as other pathogens, could greatly expand the detection spectrum and enhance the system’s ability to identify multiple pathogens simultaneously, a critical function in polymicrobial outbreaks. Additionally, experiments with more cost-effective DNA extraction methods are planned to make the technique accessible in remote areas.

Future works will be focused on the use of this technique for fleas, small mammals, and environmental samples. This expansion will not only strengthen early detection but also help monitoring animal reservoirs and vectors, which are crucial in preventing the spread of plague in endemic regions. Furthermore, evaluating this technique under real-world conditions, such as deploying mobile laboratories and testing larger sample sizes, will help validate its effectiveness and field applicability. These steps are essential for advancing towards more efficient and rapid management of plague outbreaks in remote areas and allow its implementation in these zones following the adequate biosecurity and biosafety measures.

## Supporting information

S1 Data Table of other strains used in the molecular testing. (DOCX)

S2 Data Sample’s identity, clinical form and laboratory results of plague diagnostics. (XLS)

## References

[1] Bos KI, Schuenemann VJ, Golding GB, Burbano HA, Waglechner N, Coombes BK, et al. A draft genome of *Yersinia pestis* from victims of the Black Death. Nature. 2011;478(7370):506–10. doi: 10.1038/nature10549 21993626 PMC3690193

[2] Gage KL, Kosoy MY. Natural history of plague: perspectives from more than a century of research. Annu Rev Entomol. 2005;50:505–28. doi: 10.1146/annurev.ento.50.071803.130337 15471529

[3] Anderson D, Pauling C, Beerntsen B, Song Q. Transovarial transmission of Yersinia pestis in its flea vector, Xenopsylla cheopis. Res Sq. 2023. doi: 10.21203/rs.3.rs-3397969/v1 39179552 PMC11343890

[4] Barbieri R, Signoli M, Chevé D, Costedoat C, Tzortzis S, Aboudharam G, et al. *Yersinia pestis*: the natural history of plague. Clin Microbiol Rev. 2020;34(1):e00044-19. doi: 10.1128/CMR.00044-19 33298527 PMC7920731

[5] Dennis D, Gage K, Gratz N, et al. Plague manual—epidemiology, distribution, surveillance and control. Relevé Épidémiologique Hebdomadaire. 1999. 10635759

[6] Perry RD, Fetherston JD. *Yersinia pestis*--etiologic agent of plague. Clin Microbiol Rev. 1997;10(1):35–66. doi: 10.1128/CMR.10.1.35 8993858 PMC172914

[7] Prentice MB, Rahalison L. Plague. Lancet. 2007;369(9568):1196–207. doi: 10.1016/S0140-6736(07)60566-2 17416264

[8] Pollitzer R. Plague studies. VIII. Clinical aspects. Bull World Health Organ. 1953;9(1):59–129. 13082390 PMC2542112

[9] Anderson DM, Ciletti NA, Lee-Lewis H, Elli D, Segal J, DeBord KL, et al. Pneumonic plague pathogenesis and immunity in Brown Norway rats. Am J Pathol. 2009;174(3):910–21. doi: 10.2353/ajpath.2009.071168 19164505 PMC2665751

[10] Bertherat E. Plague around the world in 2019. Wkly Epidemiol Rec. 2019;94(51–52):618–32. 31871237

[11] Chanteau S, Ratsitorahina M, Rahalison L. Current epidemiology of human plague in Madagascar. Microbes Infect. 2000;2(1):25–31. 10717537 10.1016/s1286-4579(00)00289-6

[12] Coulanges P. La peste à Madagascar (1956-1976). Arch Inst Pasteur Madag. 1978;46:397–426. 747441

[13] Andrianaivoarimanana V, Kreppel K, Elissa N, Duplantier J-M, Carniel E, Rajerison M, et al. Understanding the persistence of plague foci in Madagascar. PLoS Negl Trop Dis. 2013;7(11):e2382. doi: 10.1371/journal.pntd.0002382 24244760 PMC3820717

[14] Brygoo ER. Epidemiology of the plague at Madagascar. https://pubmed.ncbi.nlm.nih.gov/6011187. 1966. 6011187

[15] Boisier P, Rasolomaharo M, Ranaivoson G, Rasoamanana B, Rakoto L, Andrianirina Z, et al. Urban epidemic of bubonic plague in Majunga, Madagascar: epidemiological aspects. Trop Med Int Health. 1997;2(5):422–7. 9217697

[16] Rahelinirina S, Rajerison M, Telfer S, Savin C, Carniel E, Duplantier J-M. The Asian house shrew *Suncus murinus* as a reservoir and source of human outbreaks of plague in Madagascar. PLoS Negl Trop Dis. 2017;11(11):e0006072. doi: 10.1371/journal.pntd.0006072 29155827 PMC5714386

[17] Rasolomaharo M, Rasoamanana B, Andrianirina Z. Plague in Majunga, Madagascar. Lancet. 1995;346(8988):1234. 7475693 10.1016/s0140-6736(95)92944-4

[18] Vogler AJ, Chan F, Nottingham R. A decade of plague in Mahajanga, Madagascar. mBio. 2013;4(1):e00623–12. 23404402 10.1128/mBio.00623-12 PMC3573667

[19] Randremanana R, Andrianaivoarimanana V, Nikolay B, Ramasindrazana B, Paireau J, Ten Bosch QA, et al. Epidemiological characteristics of an urban plague epidemic in Madagascar, August-November, 2017: an outbreak report. Lancet Infect Dis. 2019;19(5):537–45. doi: 10.1016/S1473-3099(18)30730-8 30930106 PMC6483974

[20] D’Ortenzio E, Lemaître N, Brouat C, Loubet P, Sebbane F, Rajerison M, et al. Plague: bridging gaps towards better disease control. Med Mal Infect. 2018;48(5):307–17. doi: 10.1016/j.medmal.2018.04.393 29773334

[21] Chanteau S, Rahalison L, Ralafiarisoa L, Foulon J, Ratsitorahina M, Ratsifasoamanana L, et al. Development and testing of a rapid diagnostic test for bubonic and pneumonic plague. Lancet. 2003;361(9353):211–6. doi: 10.1016/S0140-6736(03)12270-2 12547544

[22] Baril L, Vallès X, Stenseth NC. Can we make human plague history?. BMJ Glob Health. 2019;4(4):e001984. 31799005 10.1136/bmjgh-2019-001984 PMC6861124

[23] Chanteau S. Atlas de la peste à Madagascar. 2006.

[24] Schrader C, Schielke A, Ellerbroek L, Johne R. PCR inhibitors - occurrence, properties and removal. J Appl Microbiol. 2012;113(5):1014–26. doi: 10.1111/j.1365-2672.2012.05384.x 22747964

[25] Parkhill J, Wren BW, Thomson NR, Titball RW, Holden MT, Prentice MB, et al. Genome sequence of *Yersinia pestis*, the causative agent of plague. Nature. 2001;413(6855):523–7. doi: 10.1038/35097083 11586360

[26] Woron AM, Nazarian EJ, Egan C, et al. Development and evaluation of a 4-target multiplex real-time polymerase chain reaction assay for the detection and characterization of *Yersinia pestis*. Diagn Microbiol Infect Dis. 2006;56(3):261–8. doi: 10.1016/j.diagmicrobio.2006.06.009 16949784

[27] Loïez C, Herwegh S, Wallet F, Armand S, Guinet F, Courcol RJ. Detection of *Yersinia pestis* in sputum by real-time PCR. J Clin Microbiol. 2003;41(10):4873–5. doi: 10.1128/JCM.41.10.4873-4875.2003 14532247 PMC254301

[28] Tsukano H, Itoh K, Suzuki S, Watanabe H. Detection and identification of *Yersinia pestis* by polymerase chain reaction (PCR) using multiplex primers. Microbiol Immunol. 1996;40(10):773–5. doi: 10.1111/j.1348-0421.1996.tb01140.x 8981352

[29] Tamura K, Stecher G, Kumar S. MEGA11: molecular evolutionary genetics analysis version 11. Mol Biol Evol. 2021;38(7):3022–7. doi: 10.1093/molbev/msab120 33892491 PMC8233496

[30] Stewart A, Satterfield B, Cohen M, O’Neill K, Robison R. A quadruplex real-time PCR assay for the detection of *Yersinia pestis* and its plasmids. J Med Microbiol. 2008;57(Pt 3):324–31. doi: 10.1099/jmm.0.47485-0 18287295

[31] R Core Team. R: a language and environment for statistical computing. Vienna, Austria: R Foundation for Statistical Computing; 2023. https://www.R-project.org/

[32] Bai Y, Motin V, Enscore RE, Osikowicz L, Rosales Rizzo M, Hojgaard A, et al. Pentaplex real-time PCR for differential detection of *Yersinia pestis* and Y. pseudotuberculosis and application for testing fleas collected during plague epizootics. Microbiologyopen. 2020;9(10):e1105. doi: 10.1002/mbo3.1105 32783386 PMC7568250

[33] Riehm JM, Rahalison L, Scholz HC, et al. Detection of *Yersinia pestis* using real-time PCR in patients with suspected bubonic plague. Mol Cell Probes. 2011;25(1):8–12. doi: 10.1016/j.mcp.2010.09.002 20933595

[34] Parkhill J, Wren BW, Thomson NR, Titball RW, Holden MT, Prentice MB, et al. Genome sequence of *Yersinia pestis*, the causative agent of plague. Nature. 2001;413(6855):523–7. doi: 10.1038/35097083 11586360

[35] Armougom F, Bitam I, Croce O, Merhej V, Barassi L, Nguyen T-T, et al. Genomic insights into a new *Citrobacter koseri* strain revealed gene exchanges with the virulence-associated Yersinia pestis pPCP1 plasmid. Front Microbiol. 2016;7:340. doi: 10.3389/fmicb.2016.00340 27014253 PMC4793686

[36] Rahalison L, Vololonirina E, Ratsitorahina M, Chanteau S. Diagnosis of bubonic plague by PCR in Madagascar under field conditions. J Clin Microbiol. 2000;38(1):260–3. doi: 10.1128/JCM.38.1.260-263.2000 10618097 PMC88705

[37] Eppinger M, Radnedge L, Andersen G, Vietri N, Severson G, Mou S, et al. Novel plasmids and resistance phenotypes in *Yersinia pestis*: unique plasmid inventory of strain Java 9 mediates high levels of arsenic resistance. PLoS One. 2012;7(3):e32911. doi: 10.1371/journal.pone.0032911 22479347 PMC3316555

