# Supplementary material for: Development and evaluation of a triplex real-time PCR assay for enhanced plague diagnostics in Madagascar
Source: PLoS Negl Trop Dis. 2025 Jul 24;19(7):e0013278. doi: 10.1371/journal.pntd.0013278 (PMC12313063; doi:10.1371/journal.pntd.0013278)
Supplement: S1 Data — (DOCX) [file pntd.0013278.s001.docx]

Supplementary Data S1. Table of other strains used in the molecular testing.

| **Bacterial** | **Gram Stain** | **Source** | **No. of Strain Tested** |
| --- | --- | --- | --- |
| *Escherichia coli* | Gram-negative | ZymoBioMICS | 1 |
| *Salmonella enterica* | Gram-negative | ZymoBioMICS | 1 |
| *Bartonella* sp. | Gram-negative | Plague Unit Biobank | 1 |
| *Leptospira* sp. | Gram-negative | Plague Unit Biobank | 1 |
| *Pseudomonas aeruginosa* | Gram-negative | ZymoBioMICS | 1 |
| *Rickettsia sp.* | Gram-negative | Plague Unit Biobank | 1 |
| *Cryptococcus neoformans* | Fungus-like | ZymoBioMICS | 1 |
| *Bacillus subtilis* | Gram-positive | ZymoBioMICS | 1 |
| *Clostridium difficile* | Gram-positive | Plague Unit Biobank | 1 |
| *Enterococcus faecalis* | Gram-positive | ZymoBioMICS | 1 |
| *Staphylococcus aureus* | Gram-positive | ZymoBioMICS | 1 |
| *Lactobacillus fermentum* | Gram-positive | ZymoBioMICS | 1 |
| *Listeria monocytogenes* | Gram-positive | ZymoBioMICS | 1 |
